# Evaluation of Antidiabetic Effect of Combined Leaf and Seed Extracts of *Moringa oleifera* (*Moringaceae*) on Alloxan-Induced Diabetes in Mice: A Biochemical and Histological Study

**DOI:** 10.1155/2023/9136217

**Published:** 2023-05-12

**Authors:** Badriyah Aljazzaf, Sassia Regeai, Sana Elghmasi, Nadia Alghazir, Amal Balgasim, Ismail M. Hdud Ismail, Areej A. Eskandrani, Ghalia Shamlan, Wafa S. Alansari, Ammar AL-Farga, Rabia Alghazeer

**Affiliations:** ^1^Department of Food Sciences and Nutrition, College of Health Sciences, The Public Authority for Applied Education and Training, Kuwait; ^2^Department of Life Sciences, School of Basic Science, Libyan Academy of Postgraduate Studies, Janzour, Libya; ^3^Histology and Genetics Department, Faculty of Medicine, University of Tripoli, Tripoli, Libya; ^4^Department of Biochemistry, Faculty of Medicine, University of Tripoli, Tripoli, Libya; ^5^Department of Pediatrics, Tripoli University Hospital, Faculty of Medicine, University of Tripoli, Tripoli, Libya; ^6^Biochemistry Division, Chemistry Department, Faculty of Sciences, University of Tripoli, Tripoli, Libya; ^7^Department of Pathology and Clinical Pathology, Faculty of Veterinary Medicine, University of Tripoli, Tripoli, Libya; ^8^Chemistry Department, Faculty of Science, Taibah University, Medina 30002, Saudi Arabia; ^9^Department of Food Science and Nutrition, College of Food and Agriculture Sciences, King Saud University, Riyadh 11362, Saudi Arabia; ^10^Biochemistry Department, Faculty of Science, University of Jeddah, Jeddah 21577, Saudi Arabia

## Abstract

*Moringa oleifera* (*Moringaceae*) is a medicinal plant rich in biologically active compounds. The aim of the present study was to screen *M. oleifera* methanolic leaf (L) extract, seed (S) extract, and a combined leaf/seed extract (2L : 1S ratio) for antidiabetic and antioxidant activities in mice following administration at a dose level of 500 mg/kg of body weight/day. Diabetes was induced by alloxan administration. Mice were treated with the extracts for 1 and 3 months and compared with the appropriate control. At the end of the study period, the mice were euthanized and pancreas, liver, kidney, and blood samples were collected for the analysis of biochemical parameters and histopathology. The oral administration of the combined L/S extract significantly reduced fasting blood glucose to normal levels compared with L or S extracts individually; moreover, a significant decrease in cholesterol, triglycerides, creatinine, liver enzymes, and oxidant markers was observed, with a concomitant increase in antioxidant biomarkers. Thus, the combined extract has stronger antihyperlipidemic and antioxidant properties than the individual extracts. The histopathological results also support the biochemical parameters, showing recovery of the pancreas, liver, and kidney tissue. The effects of the combined L/S extracts persisted throughout the study period tested. To the best of our knowledge, this is the first study to report on the antidiabetic, antioxidant, and antihyperlipidemic effects of a combined L/S extract of *M. oleifera* in an alloxan-induced diabetic model in mice. Our results suggest the potential for developing a natural potent antidiabetic drug from *M. oleifera*; however, clinical studies are required.

## 1. Introduction

Diabetes mellitus (DM) is one of the most common chronic diseases in the world [1–4]. DM is a health crisis of modern society and affects 537 million people around the world [5]; the population of patients with diabetes is continually increasing, and the WHO is expecting that there will by 643 million people with diabetes by the year 2030 and 783 million people by 2045 [5]. DM is a lifelong endocrine disease caused by defects in insulin secretion (i.e., deficient or insufficient synthesis of insulin from the pancreas), insulin action (i.e., insulin resistance and hyperinsulinemia), or both [6–8], leading to hyperglycemia and severe irreversible microvascular and macrovascular complications [9] that affect the eyes (diabetic retinopathy), feet (diabetic foot), nerves (diabetic neuropathy), kidneys (diabetic nephropathy), blood vessels (atherosclerosis), and heart (cardiovascular disease). The management of hyperglycemia is of utmost importance to limit the severe complications of DM [9]. The conventional treatment of DM includes insulin injections and several antidiabetic drugs such as sulfonylureas [10], metformin [11], glinides, biguanides, and acarbose [12]. Despite the success of these drugs in lowering and regulating blood glucose level, most of these antidiabetic drugs have adverse side effects, including gastrointestinal disorders, anemia, renal failure, weight gain, and hypoglycemia. Therefore, the search for new natural medications with more effective and safer properties is a priority for the discovery of new antidiabetic drugs [13–15].

Medicinal plants with low toxicity, natural antioxidants, and important bioactive phytochemicals are an excellent source of alternative natural therapies to synthetic drugs [14–17].

More than 400 herbal plants have been shown to possess antidiabetic activities, suggesting their significance for treating and managing diabetes [18–21]. Herbal plant secondary metabolites, including alkaloids, polyphenols, flavonoids, saponins, tannins, and terpenoids, have been shown to be responsible for the antihyperglycemic effect [22–24]. The reduction in glucose levels is mediated through different mechanisms, including restoring the function of pancreatic tissues by protecting the intact functional *β*-cells from further deterioration or regenerating destroyed *β*-cells, stimulating insulin secretion, inhibiting intestinal absorption of glucose, increasing insulin-induced signaling in various tissues [20, 21], and decreasing oxidative stress [25, 26].

*Moringa oleifera* is a medicinal plant of the genus *Moringa* of the family Moringaceae [27, 28]. In traditional medicine, it is known as the miracle tree [29–32] because all the plant parts (e.g., leaves, seeds, bark, roots, sap, and flowers) have nutritional and medicinal uses [28–33]. For example, the seeds possess anti-inflammatory [34], antimicrobial [35], and hepatoprotective effects [36, 37]. The preliminary phytochemical analysis of M. oleifera extracts showed the presence of simple sugar (rhamnose, isothiocyanates, and glucosinolates), niazimicin, salicylic acid, ferulic acid, vitamins (for example, ascorbic acid), provitamins (such as tocopherols and carotenoids), minerals (such as potassium and calcium), and “secondary metabolites” (including phenols, flavonoids, tannins, and alkaloids) [38]. The leaves of *M. oleifera* have high nutritional value; they are rich in calcium, protein, and vitamins A, B, and C [31–35, 37, 39, 40]. The leaves are also used to treat wounds, fever, sores, bronchitis, eye, and ear infections [41]. Moreover, several studies have shown that the aqueous extracts of *M. oleifera* leaves possess a wide range of biological actions including antioxidant, tissue protective, cardioprotective, hepatoprotective, neuroprotective [42, 43], analgesic [44], diuretic [45], antiulcer [46], anticancer [18], antidiabetic, anti-inflammatory [47], antimicrobial [48], antihypertensive [49], radioprotective [50, 51], and immunomodulatory [52, 53] effects. Furthermore, it was reported that the administration of an aqueous extract of *M. oleifera* leaves or seeds manifested potent antihyperglycemic [54, 55] and antihyperlipidemic [39, 56] effects in insulin-resistant and insulin-deficient rat models [30, 57]. Therefore, the present study was undertaken to perform an experimental validation of the antihyperglycemic, antihyperlipidemic, and antioxidative roles of *M. oleifera* leaf extract, seed extract, and a combination of the leaf and seed extracts. To the best of our knowledge, there are no published studies that have used the combination (i.e., mixture) of leaf and seed extracts of *M. oleifera* to investigate its antihyperglycemic (antidiabetic) effects.

The objective of this study was to investigate the antidiabetic effect of the methanolic extracts of *M. oleifera* leaf, seed, and their combination following administration at a dose level of 500 mg/kg of body weight (kg BW)/day. Diabetes was induced in mice using alloxan, which is a well-established method [58]. Alloxan induces diabetes by damaging the insulin-secreting pancreatic *β*-cells, resulting in a decrease in endogenous insulin release and a decrease in glucose utilization by the tissues, which leads to hyperglycemia [58, 59].

## 2. Material and Methods

### 2.1. Chemicals

Alloxan monohydrate was obtained from Sigma-Aldrich Chemical Company (St. Louis, MA, USA). 5,5′-Dithiobis (2-nitrobenzoic acid) (DTNB) was obtained from BDH Chemicals (England). Methanol, ethanol, chloroform, guanidine hydrochloride, 2,4-dinitrophenylhydrazine (DNPH), trichloroacetic acid (TCA), thiobarbituric acid (TBA), and hydrochloric acid were obtained from Merck (Pvt.) Ltd. (Germany). Phosphate buffered saline (PBS), hydrogen peroxide, and 1,1,3,3-tetramethoxypropane (TMP) were obtained from Sigma Chemical Company Ltd. (USA). All solvents and other reagents were of standard analytical grade. Commercial kits for biochemical analysis of liver enzymes (aspartate aminotransferase (AST), alanine aminotransferase (ALT), and alkaline phosphate (ALP)), total protein, and creatinine were obtained from Biomagrab, Tunisia.

### 2.2. Collection of Plant Material

**Figure 1:**
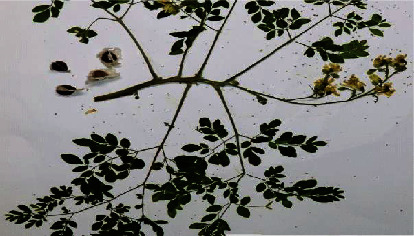
*Moringa oleifera* (*Moringaceae*) leaves and seeds collected from Tajoura, Tripoli, Libya.

Fresh *Moringa Oleifera* (*Moringaceae*) (Figure 1) leaves and seeds were collected from Mohamed Alkamoushi garden in Tajoura, Tripoli, Libya. The identity of the plant was confirmed by Dr. M. Abohadra, a plant taxonomist at Botany Department, Faculty of Science, University of Tripoli. A voucher specimen of *M. oleifera* (FHI-110287) was deposited in the herbarium at the Faculty of Science, University of Tripoli.

### 2.3. Preparation of Crude Leaf and Seed Extracts

Freshly collected leaves and seeds were air-dried in the shade at room temperature and then ground into powder. The dried powder was then stored at 4°C until used. Crude extracts were prepared from 25 g of *M. oleifera* leaf or seed powder soaked in 500 mL of methanol for 72 h with gentle shaking. The methanol extract was filtered using Whatman filter paper No. 1; then, the methanol was removed using a rotary evaporator to obtain the pure crude stock extract. The yield of methanol extracts was 3.8% for leave and 2.8% for seeds. The leaf or seed stock extracts were stored at 4°C until use [60]. Distilled water was used as the diluent to reconstitute the extract and administered orally for 1 or 3 months. In our preliminary experiments, the dose of 500 mg/kg body weight/day of *M. oleifera* was found to be the most suitable treatment.

### 2.4. Qualitative Phytochemical Analysis

The extracts were subjected to phytochemical screening as described by Harborne [61].

### 2.5. Quantitative Determination of Phytochemicals

Total phenolic content was evaluated according to the Folin–Ciocalteu method by Singleton et al. [62] using gallic acid as the standard. Total flavonoid content was evaluated as described by Zhishen et al. [63] using rutin as the standard. Total alkaloid content was determined as described by [64] using atropine as the standard. The total anthocyanidin content was determined using the vanillin-HCl colorimetric method [65] using catechin as the standard.

### 2.6. Ethical Approval

All experiments in this study complied with the bioethical research established by the Libyan National Committee for Biosafety and Bioethics, Biotechnology Research Center, University of Tripoli (Reference BEC-BTRC 7-2019), and the methodology used conforms to the published principles of laboratory animal care [66].

### 2.7. Animals

Adult male Swiss albino mice, 8–10 weeks old and weighing 25–30 g, were used in this study. These mice were obtained from the animal house of the Chemistry Department, Faculty of Science, University of Tripoli. Polycarbonate cages with wood shavings for bedding and steel wire tops were used to house three mice per cage. Mice were kept under appropriate conditions and allowed free access to food and water. Mouse body weight was measured every week using a digital balance to determine changes in each experimental animal.

### 2.8. Experimental Induction of Diabetes

Diabetes was induced by alloxan administration for all mice except for the normal control group (group 1). Diabetes induction comprised a single intraperitoneal injection of 200 mg/kg of alloxan monohydrate [59] following an overnight fast. Diabetes was confirmed via the analysis of blood from mouse tail by glucose oxidase method using a glucometer (One Basic, Inc.) at 72 h after alloxan injection. A plasma glucose level of ≥200 mg/dL indicated hyperglycemia in alloxan-treated mice. These mice were used as diabetic animal models in this study.

### 2.9. Toxicity Test

The acute toxicity test was performed in accordance with OECD 423 guidelines (Organization for Economic Co-operation and Development, Guideline 423) [67]. The acute oral toxicity of extracts of *M. oleifera* leaf, seed, and their mixture was evaluated in mice at doses of 200, 400, 500, 1000, 2000, and 5000 mg/kg BW. The treatment was either a single large dose (2000 and 5000 mg/kg BW) or a daily small dose (200 mg/kg BW/day) for 1 month. The value of oral lethal dose (LD_50_) of the *M. oleifera* in this study was greater than 2000 mg/kg BW in mice. Distilled water was used as the diluent for reconstituting the extract and administered orally. Twice-daily observations of the mice were performed throughout the study period, and signs of behavioral changes and/or mortality were recorded.

### 2.10. Experimental Groups

The test mice were divided into nine treatment groups with three mice in each group (Table 1). Group (G)1 comprised nondiabetic control mice who did not receive any treatment; G2 comprised diabetic control mice who did not receive any treatment; G3 comprised diabetic mice treated with 0.7 units/kg insulin; groups 4, 5, and 6 comprised diabetic mice treated with extracts (500 mg/kg BW/day) of *M. oleifera* leaf extract (G4), seed extract (G5), and the combination extract (G6) for 1 month. Groups 7, 8, and 9 comprised diabetic mice treated for 3 months with extracts (500 mg/kg BW/day) of *M. oleifera* leaf extract (G7), seed extract (G8), and the combination extract (G9).

At the end of the study period and 24 h after the last treatment, the mice were anesthetized by chloroform. After blood samples were collected, the mice were euthanized by cervical dislocation. Then, the liver, kidney, and pancreas were collected. A portion of each tissue was used to prepare a 10% tissue homogenate to determine the biochemical parameters; the remainder was preserved in 10% formalin for histopathological examination.

### 2.11. Blood Glucose Determination

Blood samples were collected after overnight fasting from all experimental groups by tail puncture method, and blood glucose levels were determined on days 0, 7, 14, 21, 28, and 90 using a glucometer (One Basic, Inc.).

### 2.12. Serum and Homogenate Preparation

The blood was collected from the heart, centrifuged at 3000 rpm for 5 min to obtain serum, and stored at −20°C for biochemical analysis [68]. Separately, 10% weight/volume (*w*/*v*) liver and kidney homogenates were prepared in ice-cold PBS using a homogenizer (IKA, 20.n, Germany). The homogenates were centrifuged at 3000 rpm for 10 min at 4°C. The homogenate supernatant was separated in aliquots and stored at −20°C until biochemical analysis. The homogenate was used for the determination of lipid peroxidation levels and the activity of antioxidant enzymes.

### 2.13. Estimation of Biochemical Parameters in Serum

The estimation of serum aspartate aminotransferase (AST), alanine aminotransferase (ALT), alkaline phosphatase (ALP), creatinine, cholesterol (TC), and triglyceride (TG) was conducted in accordance with the manufacturer's protocol (Biomagrab, Tunisia). The total protein content in the serum was assayed using the Biuret method [69].

### 2.14. Determination of Oxidative Stress and Antioxidant Biomarker Activity

In this study, oxidative stress and antioxidant biomarkers in the liver and kidney homogenates included lipid peroxidation, nitric oxide (NO), protein carbonyl (PC), reduced glutathione (GSH), and catalase (CAT) activities. Lipid peroxidation levels were determined spectrophotometrically at 353 nm [70] from the reaction of malondialdehyde (MDA) with thiobarbituric acid conjugate, and the amount of MDA was expressed as nmol/mg protein. Nitric oxide was determined spectrophotometrically at 546 nm [71]. The PC content was measured spectrophotometrically at 370 nm [72]. The levels of GSH were measured through spectrophotometric measurement of 5-thiol-2-nitrobenzoic acid formation at 412 nm and expressed as *μ*mol/mg of protein. CAT activity was assayed as described by the method of [73]. The CAT activity was measured calorimetrically at 570 nm.

### 2.15. Histological Examination

The liver, kidney, and pancreas were rapidly excised, washed by normal cold saline, blotted dry, and immediately weighed. The preserved tissues were dehydrated, cleared, and embedded in paraffin wax [74]. Tissue blocks were sectioned at a thickness of 7 *μ*m and stained with hematoxylin and eosin. The slides were examined under a light microscope for the histopathological investigation and photographed at 400x magnification.

### 2.16. Statistical Analysis

The results were expressed as the mean ± standard deviations (SD) of triplicate determinations. All statistical analyses and graphs were performed using SPSS version 24. Statistical comparisons between the experimental groups were performed using one-way analysis of variance (ANOVA), to test for any differences between mean values of the treated groups compared with those of the control group, followed by Tukey's multiple comparison posttest. Differences were considered statistically significant when *P* < 0.05.

## 3. Results

### 3.1. Qualitative and Quantitative Phytochemical Analyses

In the current study, the preliminary qualitative phytochemical analysis was performed for leaves and seeds of *Moringa oleifera.* The results revealed that leaf extract showed presence of polyphenols, flavonoids, tannins, alkaloids, saponins, coumarins, and terpenoids, while seed extract showed absence of tannins and terpenoids (Table 2).

The contents of total phenolics, total flavonoids, anthocyanidin, and alkaloids are presented in Table 3. The leaf extract has higher phenolic content, flavonoids, anthocyanidin, and alkaloids than the seed extract which elucidates the reason for the strong and efficient antioxidant properties.

### 3.2. Toxicity of *Moringa oleifera*

The different tested doses of extracts of *M. oleifera* leaf, seed, or combination extracts caused no adverse effects in all experimental animals. Mortality was the main criterion for the assessment of acute toxicity. No mortality and no toxicity were observed in the treated mice. These results confirm that the *M. oleifera* leaf, seed, and combination extracts were safe.

### 3.3. Effect of *Moringa oleifera* on Blood Glucose Level

**Figure 2:**
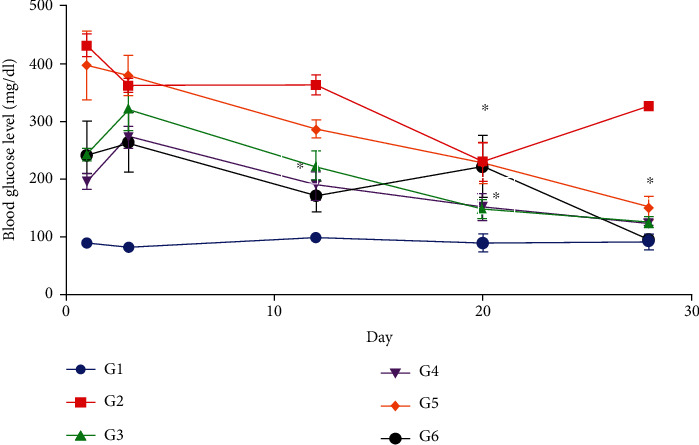
Blood glucose levels in nondiabetic control mice (G1, blue line), diabetic control mice (G2, red line), insulin-treated diabetic mice (G3, green line), *Moringa oleifera* leaf extract (500 mg/kg BW/day)-treated diabetic mice (G4, purple line), *Moringa oleifera* seed extract (500 mg/kg BW/day)-treated diabetic mice (G5, orange line), and *Moringa oleifera* leaf/seed (ratio 2L : 1S) combination-treated diabetic mice (G6, black line). ^∗^ indicates significant change compared with the diabetic group (G2) (*P* < 0.05) by one-way ANOVA followed by Tukey's test.

All mice in the alloxan-treated groups (G2 to G6) developed severe diabetes, as indicated by an increase in plasma glucose levels, from 226.3 ± 6.3 to 431.7 ± 20.0 mg/dL, from day 1 of the experiment. However, the administration of a daily dose of insulin (G3) or *M. oleifera* leaf (G4), seed (G5), and combination (G6) extracts resulted in a significant decrease in blood glucose levels compared to the diabetic control (G2) mice throughout the study period (Figure 2). The highest decrease in blood glucose level was observed in G6 after the administration of combination of leaf and seed extracts of *M. oleifera* followed by the administration of leaf extract (G4) (Figure 2). This decrease was greater than observed after insulin treatment (G3). In addition, there was a mild decrease in blood glucose level after treatment with seed extract of *M. oleifera* (G5). After 1 month of daily treatment with insulin (G3) or *M. oleifera* leaf (G4), seed (G5), and combination (G6) extracts, a significant reduction in blood glucose levels was observed compared with the diabetic control group (G2); blood glucose was near normal level compared with the normal control mice (G1) (Figure 2).

Moreover, after 3 months of daily treatment with insulin (G3) or an extract of *M. oleifera*, blood glucose level decreased significantly to 107.3 ± 12.0 mg/dL in the leaf extract-treated group (G4), 113.6 ± 20.55 mg/dL in the seed extract-treated group (G5), and 95.7 ± 8.6 mg/dL in the combination extract-treated group (G6) compared with the diabetic groups on day 1 of treatment.

### 3.4. Effect of *Moringa oleifera* on Body Weight

**Figure 3:**
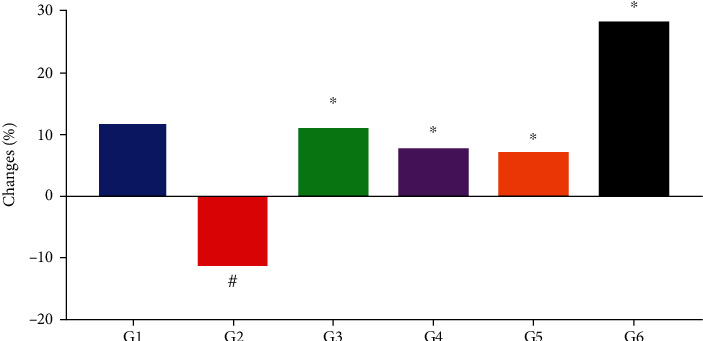
The effect of *Moringa oleifera* on body weight changes (%) in the experimental groups. G1, nondiabetic control mice; G2, diabetic control mice; G3, insulin-treated diabetic mice; G4, *Moringa oleifera* leaf extract (500 mg/kg BW/day)-treated diabetic mice; G5, *M. oleifera* seed extract (500 mg/kg BW/day)-treated diabetic mice; G6, *Moringa oleifera* combination extract-treated diabetic mice. ^∗^ indicates significant change compared with the diabetic group (G2) (*P* < 0.01), and # indicates significant change compared with the nondiabetic group (G1) (*P* < 0.01) by one-way ANOVA followed by Tukey's test.

The results of this study revealed differences in body weight changes between the experimental groups. There were observable body weight changes in the alloxan-induced diabetic mice. The decrease in the body weight of the diabetic control mice (G2) was significant (*P* < 0.05) compared to that of the nondiabetic control mice (G1) and experimental groups G3, G4, and G5 (Figure 3). By the end of the study period, the diabetic mice (G2) had lost approximately 11.5% of their body weight (Figure 3). However, this significant weight loss was prevented in diabetic mice that were treated with insulin or *M. oleifera* leaf, seed, and combination extracts. The approximate body weight loss in the different experimental groups was 0.9% in the insulin-treated group (G3) and 3.1% and 3.6% in *M. oleifera* leaf (G4)- and seed (G5)-treated groups, respectively (Figure 3) and 16.4% in the *M. oleifera* combination extract-treated group (G6), which was a significant difference (*P* < 0.01) (Figure 3).

### 3.5. Effect of Moringa Oleifera on Pancreas, Kidney, and Liver Weights

**Figure 4:**
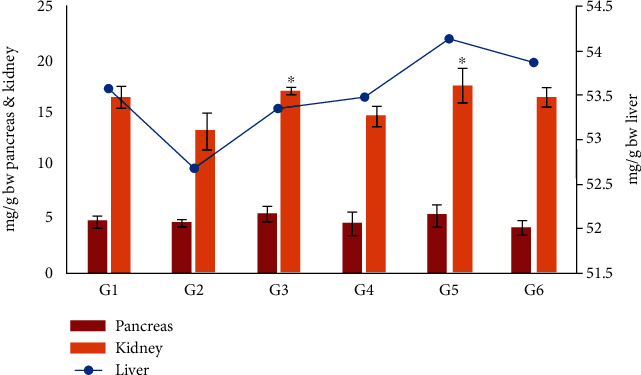
The effect of *Moringa oleifera* on the weight of the pancreas, kidney, and liver in the different experimental groups. G1, nondiabetic control mice; G2, diabetic control mice; G3, insulin-treated diabetic mice; G4, *Moringa oleifera* leaf extract (500 mg/kg BW/day)-treated diabetic mice; G5, *M. oleifera* seed extract (500 mg/kg BW/day)-treated diabetic mice; G6, *Moringa oleifera* combination extract-treated diabetic mice. Values are expressed as the mean ± SD. ^∗^ indicates significant change compared with nondiabetic control (*P* < 0.01) by one-way ANOVA followed by Tukey's test.

The weights of the pancreas, kidney, and liver in the different experimental groups are shown in Figure 4. The organ weights were significantly higher in the groups treated with insulin (G3) or *M. oleifera* leaf (G4), seed (G5), and combination (G6) extracts compared with the diabetic control group (G2) (*P* < 0.05) (Figure 4). The largest increase in organ weight was recorded in mice treated with the *M. oleifera* combination extract, where the weight index of the pancreas, kidney, and liver decreased to 4.7 ± 1.1, 16.2 ± 0.96, and 53.8 ± 1.2 mg/g BW, respectively.

### 3.6. Effect of *Moringa oleifera* on Liver Enzyme Activity

**Figure 5:**
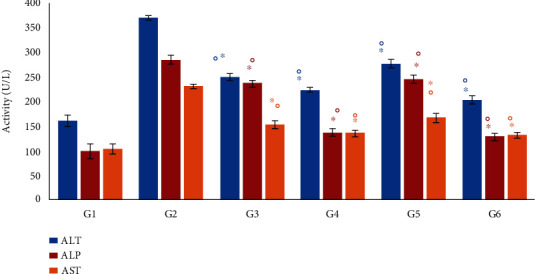
The effect of *Moringa oleifera* on activities of the liver enzymes ALT, ALP, and AST in the experimental groups. G1, nondiabetic control mice; G2, diabetic control mice; G3, insulin-treated diabetic mice; G4, *Moringa oleifera* leaf extract (500 mg/kg BW/day)-treated diabetic mice; G5, *M. oleifera* seed extract (500 mg/kg BW/day)-treated diabetic mice; G6, *Moringa oleifera* combination extract-treated diabetic mice. Values are expressed as the mean ± SD. ° indicates significant change compared with the diabetic control (G2) group (*P* < 0.01), and ^∗^ indicates significant change compared with the nondiabetic control (G1) group (*P* < 0.01), both by one-way ANOVA followed by Tukey's test.

The activities of liver enzymes (aspartate aminotransferase (AST), alanine aminotransferase (ALT), and alkaline phosphatase (ALP)) in serum were increased in all diabetic mice (Figure 5), indicating hepatic damage and the leaking of enzymes from the tissues into the circulation as an adverse effect of alloxan-induced diabetes. This harmful effect was significantly reversed following administration of *M. oleifera* leaf (G4), seed (G5), and combination (G6) extracts (Figure 5), where a significant (*P* < 0.01) reduction in liver enzyme activities was observed in all treated diabetic mice compared with the corresponding diabetic control (G2). In addition, the most significant (*P* < 0.01) decrease in liver enzyme activities (AST, ALT, and ALP) was seen in G6, where the values decreased to values closer to the normal levels found in the nondiabetic control G1 (Figure 5).

### 3.7. Effect of *Moringa oleifera* on Serum Creatinine Level

**Figure 6:**
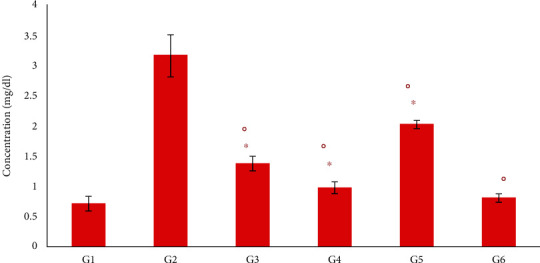
Effect of *Moringa oleifera* on serum creatinine levels in the experimental groups. G1, nondiabetic control mice; G2, diabetic control mice; G3, insulin-treated diabetic mice; G4, *Moringa oleifera* leaf extract (500 mg/kg BW/day)-treated diabetic mice; G5, *M. oleifera* seed extract (500 mg/kg BW/day)-treated diabetic mice; G6, *Moringa oleifera* combination extract-treated diabetic mice. Values are expressed as the mean ± SD. ° indicates significant change compared with the diabetic control group (*P* < 0.01), and ^∗^ indicates significant change compared with nondiabetic control (*P* < 0.01) by one-way ANOVA followed by Tukey's test.

In the current study, a substantial increase in creatinine levels was observed in the diabetic groups compared with the nondiabetic control group (G1) (*P* < 0.01) (Figure 6). However, there was a significant reduction (*P* < 0.05) in the level of creatinine in the groups treated with insulin or *M. oleifera* extracts compared with the diabetic control group (G2). The maximum reduction (*P* < 0.05) in creatinine level was observed in mice treated with *M. oleifera* combination extract (G6) (Figure 6).

### 3.8. Effect of *Moringa oleifera* on Total Cholesterol and Triglycerides

**Figure 7:**
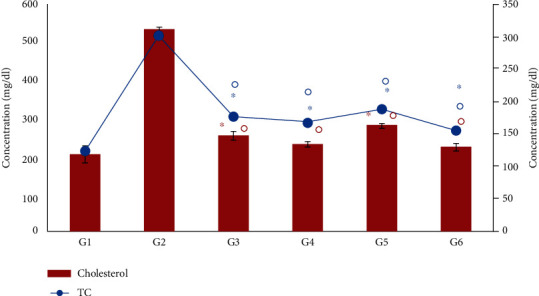
Effect of *Moringa oleifera* on the total cholesterol and triglyceride levels in the experimental groups. G1, nondiabetic control mice; G2, diabetic control mice; G3, insulin-treated diabetic mice; G4, *Moringa oleifera* leaf extract (500 mg/kg BW/day)-treated diabetic mice; G5, *M. oleifera* seed extract (500 mg/kg BW/day)-treated diabetic mice; G6, *Moringa oleifera* combination extract-treated diabetic mice. Values are expressed as the mean ± SD. ° indicates significant change compared with the diabetic control group (*P* < 0.01) and ^∗^ indicates significant change compared with the normal control (*P* < 0.01) by one-way ANOVA followed by Tukey's test.

Serum cholesterol (CT) and triglycerides (TG) were significantly (*P* < 0.01) increased in the diabetic control group (G2) with respect to the nondiabetic control group (G1) (Figure 7). The cholesterol levels were substantially reduced (*P* < 0.001) to 254.33 ± 12.0, 231 ± 7.0, 281.33 ± 7.5, and 223.67 ± 11.1 mg/dL by insulin, leaf, seed, and combination extracts, respectively, compared with 533.3 ± 7.02 mg/dL in the diabetic control (G2) (Figure 7). In contrast, the triglyceride level was decreased significantly (*P* < 0.001) to 177.67 ± 6.5, 169 ± 7.6, 190.33 ± 19.6, and 154 ± 5.6 mg/dL, by insulin, leaf, seed and combination extracts, respectively, compared with 302.7 ± 13.1 mg/dL in the diabetic control group (G2) (Figure 7). The maximum reduction (*P* < 0.05) in the amount of cholesterol and triglyceride levels was observed in the *M. oleifera* combination-treated group (G6), indicating significant recovery from alloxan-induced diabetes.

### 3.9. Effect of *Moringa oleifera* on Oxidative Stress Biomarkers in the Liver and Kidney

**Figure 8:**
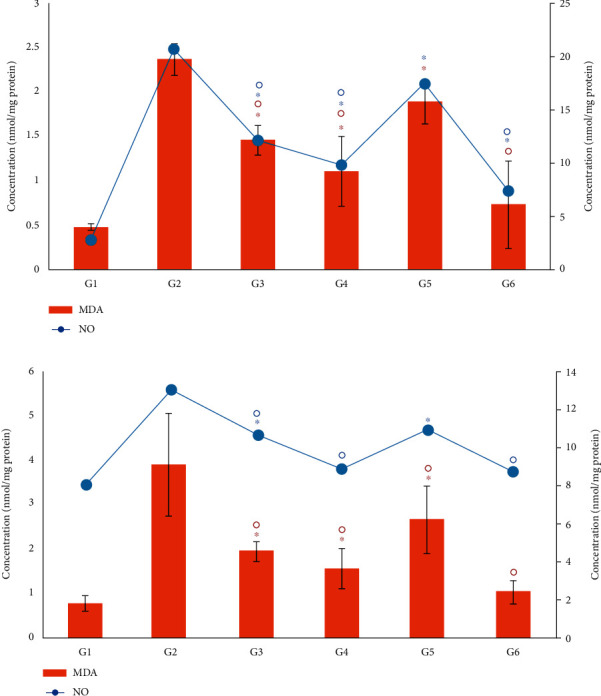
Effect of *Moringa oleifera* on the oxidative stress biomarkers MDA and NO in the liver (a) and kidney (b) tissue homogenate in the experimental groups. G1, nondiabetic control mice; G2, diabetic control mice; G3, insulin-treated diabetic mice; G4, *Moringa oleifera* leaf extract (500 mg/kg BW/day)-treated diabetic mice; G5, *M. oleifera* seed extract (500 mg/kg BW/day)-treated diabetic mice; G6, *Moringa oleifera* combination extract-treated diabetic mice. Values are expressed as the mean ± SD. ° indicates significant change compared with the diabetic control group (*P* < 0.01), and ^∗^ indicates significant change compared with the nondiabetic control (*P* < 0.01) by one-way ANOVA followed by Tukey's test.

**Figure 9:**
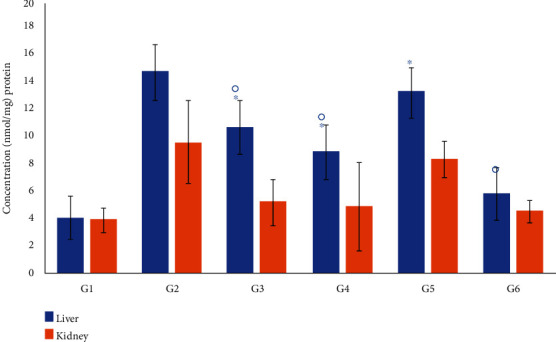
Effect of *Moringa oleifera* on the oxidative stress biomarker, protein carbonyl (PC), in the liver and kidney tissue homogenates in the experimental groups. G1, nondiabetic control mice; G2, diabetic control mice; G3, insulin-treated diabetic mice; G4, *Moringa oleifera* leaf extract (500 mg/kg BW/day)-treated diabetic mice; G5, *M. oleifera* seed extract (500 mg/kg BW/day)-treated diabetic mice; G6, *Moringa oleifera* combination extract-treated diabetic mice. Values are expressed as the mean ± SD, one-way ANOVA followed by Tukey's test. ° indicates significant change compared with the diabetic control group (*P* < 0.01), and ^∗^ indicates significant change compared with nondiabetic control (*P* < 0.01) by one-way ANOVA followed by Tukey's test.

The levels of MDA, NO, and PC as oxidative stress biomarkers in the liver and kidney are presented in Figure 8. In the diabetic control group (G2), the levels of MDA, NO, and PC in the tested tissues were significantly (*P* < 0.05) increased compared with their corresponding tissues in the nondiabetic control (G1) (Figures 8 and 9). The levels of MDA, NO, and PC returned closer to normal values after treatment with insulin or *M. oleifera* extracts. Treatment of diabetic mice with the *M. oleifera* combination extract significantly reduced (*P* < 0.05) the level of MDA in the liver and kidney by 68.8% and 73.3%, respectively, which decreased the levels of NO by 64.0% in the liver and 33.0% in the kidney. The levels of PC were also greatly reduced (*P* < 0.05) in the liver (60%) and kidney (52%) compared with the diabetic control G2 (Figures 8 and 9).

**Figure 10:**
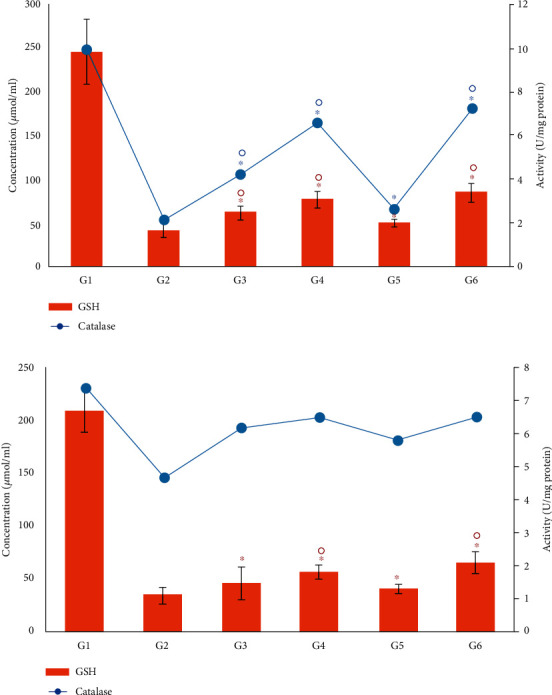
Effect of *Moringa oleifera* on the antioxidant biomarkers, GSH and catalase, in the liver (a) and kidney (b) tissue homogenates in the experimental groups. G1, nondiabetic control mice; G2, diabetic control mice; G3, insulin-treated diabetic mice; G4, *Moringa oleifera* leaf extract (500 mg/kg BW/day)-treated diabetic mice; G5, *M. oleifera* seed extract (500 mg/kg BW/day)-treated diabetic mice; G6, *Moringa oleifera* combination extract-treated diabetic mice. Values are expressed as the mean ± SD. ° indicates significant change compared with the diabetic control group (*P* < 0.01), and ^∗^ indicates significant change compared with the nondiabetic control (*P* < 0.01) by one-way ANOVA followed by Tukey's test.

In the present study, the changes in the catalase activity and GSH level in the liver and kidney of the nondiabetic control (G1) and diabetic groups were measured (Figure 10). In the diabetic control group (G2), a large decrease in the level of GSH and the activity of CAT in the kidney and liver tissues was found. However, the levels of GSH and catalase activity were increased after the administration of the *M. oleifera* combined extract-treated group by 51.6% (liver) and 46.7% (kidney) for GSH and by 70.3% (liver) and 28.5% (kidney) for CAT activity (Figure 10).

**Figure 11:**
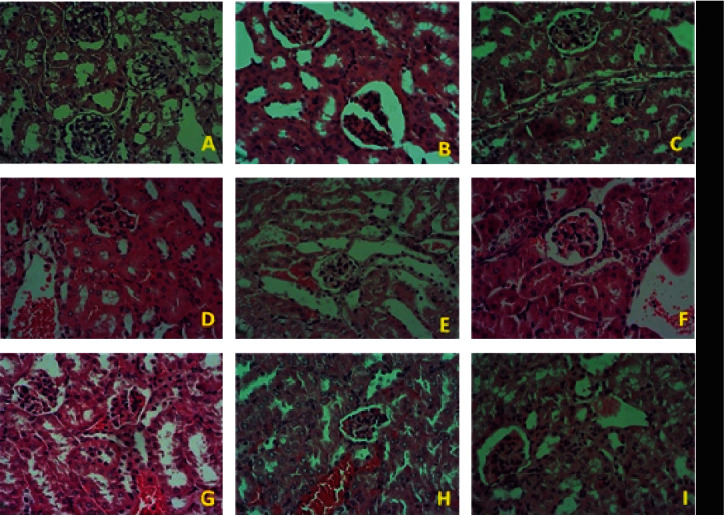
Histological sections of kidney in the experimental groups. (a) Renal tissue of nondiabetic control mice (G1) with normal histological structure of the renal parenchyma, Bowman's capsule, glomeruli, and kidney tubules. (b) Kidney tissue of diabetic control mice (G2) showing renal histopathological changes that include vacuolation of the endothelial lining of the glomerular tuft and of the epithelial lining renal tubules. (c) Insulin-treated diabetic mice (G3) showing normal renal tissue structure with no histopathological changes. (d–f) The renal tissue of diabetic mice treated with 500 mg/kg BW/day *Moringa oleifera* leaf (G4), seed (G5), and combination (G6) extracts, respectively, for 1 month, where mild histopathological changes persisted. (g–i) The renal tissue of diabetic mice treated with 500 mg/kg BW/day *Moringa oleifera* leaf (G7), seed (G8), and combination (G9) extracts, respectively, for 3 months, with a noticeable decrease in renal histopathological changes (hematoxylin and eosin stained, 400x magnification).

**Figure 12:**
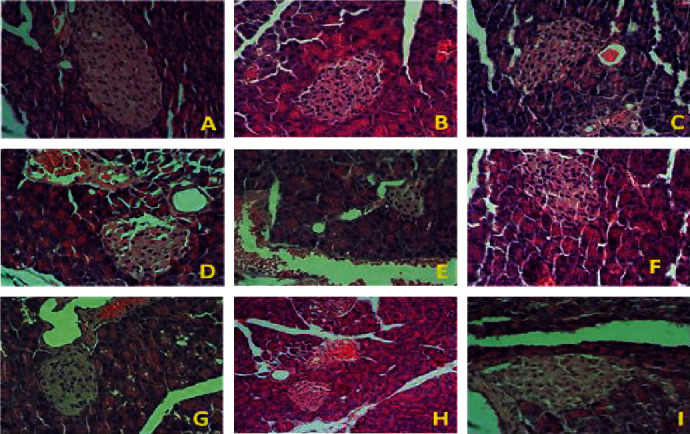
Histological sections of the pancreas in the experimental groups. (a) Pancreatic tissue of nondiabetic control mice (G1) showing the normal histological structure of the pancreas parenchyma; pancreatic lobules are surrounded by connective tissue capsules, excretory ducts, regular islets of Langerhans, and secretory acini. (b) Pancreatic tissue of diabetic control mice (G2) showing pancreatic histopathological changes that include irregular pancreatic acini tissue, severe shrinkage and necrosis of islet of Langerhans with fatty infiltration in the pancreas parenchyma, severe congestion of blood vessels, and vacuolations of pancreatic acini, disrupted outlining of islets of Langerhans, and dilated intralobular ducts. (c) Insulin-treated diabetic mice (G3) showing mild pancreatic histopathological changes. (d, e) Pancreatic tissue of diabetic mice treated with 500 mg/kg BW/day *Moringa oleifera* leaf (G4) and seed (G5) extracts for 1 month with persistent histopathological changes: shrinkage in the islets of Langerhans and less fatty infiltration. (g–i) Pancreatic tissue of diabetic mice treated with extracts (500 mg/kg BW/day) of *Moringa oleifera* leaf (G7), seed (G8), and combination (G9) extracts for 3 months, showing the significant recovery of pancreatic histopathological changes, especially in G6 and G9 (f, i), which show the normal arrangement of pancreatic acini and islets of Langerhans, similar to the nondiabetic control group (G1) (hematoxylin and eosin staining, 400x magnification).

**Figure 13:**
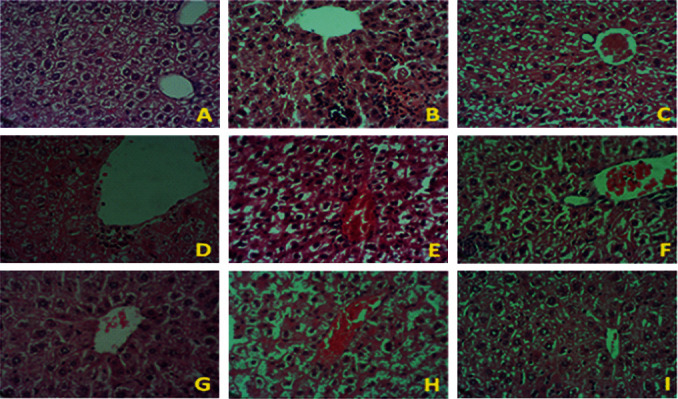
Histological sections of liver samples from the different experimental groups. (a) Hepatic tissue of nondiabetic control mice (G1) showing the normal histological structure of the hepatic parenchyma, hepatic lobules comprised of hepatocytes, the central vein, and the portal vein. Hepatocytes arranged in cords radiating from the central vein toward the portal area. The gaps between the hepatic cords comprise the sinusoids. (b) Hepatic tissue of diabetic control mice (G2) showing hepatic histopathological changes that include the accumulation of fat vacuoles in hepatocytes, mild inflammatory cell infiltration, vascular dilatation and congestion, and varying degree of fibrosis at the pericellular and perisinusoidal levels. In the central area, degenerative changes characterized by vacuolation of hepatocytes in the central zone area of the hepatic lobule. (c) Insulin-treated diabetic mice (G3) showing mild changes in hepatic histopathology. (d–f) Hepatic tissue of diabetic mice treated with 500 mg/kg BW/day of *Moringa oleifera* leaf (G4), seed (G5), and combination (G6) extracts, respectively, for 1 month, exhibiting persistent histopathological changes, including degeneration and vacuolization in hepatocytes. (g–i) Hepatic tissue of diabetic mice treated with 500 mg/kg BW/day of *Moringa oleifera* leaf (G7), seed (G8), and combination (G9) extracts, respectively, for 3 months, illustrating significant improvement in the hepatic histoarchitecture, especially in the G9 group, which shows lower congestion and less emptying of hepatocytes (i). Hematoxylin and eosin staining, 400x magnification.

Additionally, alloxan-induced diabetic mice were treated for 3 months with *Moringa oleifera* leaf (G7), seed (G8), and combination (G9) extracts. These groups were monitored for 3 months to confirm the persistent antidiabetic and antioxidant effects of *M. oleifera* extracts on biochemical parameters (Table 4) and the histology of the kidney, pancreas, and liver (Figures 11–13). Table 4 shows the results for persistent lower levels of lipid profile, triglyceride (TG), cholesterol (TC), liver enzymes (alkaline phosphatase (ALP), aspartate aminotransferase AST (AST), and alanine aminotransferase (ALT)), and oxidative stress biomarkers (malondialdehyde (MDA), nitric oxide (NO), and protein carbonyl (PC)), as well as persistent increase in antioxidant activities of reduced glutathione (GSH) and catalase (CAT) compared with diabetic control (G2) and diabetic-treated mice for 1 month with *Moringa oleifera* leaf (G4), seed (G5), and combination (G6) extracts. These findings were confirmed by the antidiabetic activity of tested samples, as well as the amelioration of histopathological changes.

### 3.10. Histological Effect of *Moringa oleifera* on the Kidney, Liver, and Pancreas Tissues

Microscopic examination of the kidney of nondiabetic control mice (G1) shows normal renal histology architecture, comprised of intact Bowman's capsule, glomeruli, glomerular tuft surrounded by glomerular space, and renal tubules lined by epithelial cells (Figure 11(a)). However, microscopic examination of the kidney from the diabetic control group (G2) revealed the abnormal histology of renal parenchymal architecture, which includes vacuolation of the endothelial lining glomerular tuft and of the epithelial lining of renal tubules, glomerular atrophy and segmentation, severe infiltration of inflammatory cells, congested blood vessels, renal tubules exhibiting moderate cellular swelling, vacuolation, necrosis, and sloughing of some endothelial cells (Figure 11(b)). The kidneys of diabetic mice treated with insulin (G3) show normal histological renal parenchymal architecture (Figure 11(c)). In contrast, diabetic mice treated with *M. oleifera* leaf (G4), seed (G5), and combination (G6) extracts for 1 month did not significantly improve renal tissue damage caused by diabetes (Figures 11(d)–11(f)); in contrast, the extended treatment of diabetic mice with *M. oleifera* leaf (G7), seed (G8), and combination (G9) extracts for 3 months restored the renal structure to a noticeably normal histology in diabetic mice (Figures 11(g)–11(i)).

The histological examination of the pancreatic tissue of normal and diabetic mice in the present study is shown in Figure 12. Figure 12(a) displays the normal structure of the pancreas in the nondiabetic control group (G1) where the pancreatic lobules are surrounded by connective tissue capsule containing blood vessels, nerves, lymphatics and excretory ducts, and regular islets of Langerhans with clustered *β*-cells located centrally. The secretory acini are composed of tubular and spherical masses of cells. The islets of Langerhans consist of anastomosing cords of polygonal endocrine cells (Figure 12(a)). However, the pancreatic tissue of all diabetic mice groups present histopathologic damage, including fatty changes in the pancreas parenchyma, severe shrinkage and necrosis of islet of Langerhans, with severe congestion of blood vessels, and vacuolations of the pancreatic acini (Figures 12(b)–12(i)). Furthermore, the treatment of diabetic mice with 500 mg/kg BW/day *M. oleifera* leaf (G4, Figure 12(d)) or seed (G5, Figure 12(e)) extracts for 1 month or 3 months G7 (Figure 12(g)) or G8 (Figure 12(h)) did not improve the histopathological changes present in the pancreas of diabetic mice (Figures 12(d), 12(e), 12(g), and 12(h)). However, the treatment of diabetic mice with *M. oleifera* combination extract for 1 month, G6 (Figure 12(f)), or 3 months, G9 (Figure 12(i)), resulted in significant recovery of histopathological changes in the pancreas, as indicated by normal acini and normal islets of Langerhans, similar to those found in the normal nondiabetic control group, G1 (Figure 12(a)).

The liver histopathological findings from nondiabetic and diabetic mice are shown in Figure 13. Figure 13(a) illustrates the hepatic tissue of the nondiabetic control group (G1), showing normal liver architecture, hepatic lobules comprised of hepatocytes, the central vein, the hepatic artery, the portal vein, and bile ducts. Hepatocytes arranged in cords radiating from central vein toward the portal area. The gaps between hepatic cords comprised the sinusoids. Figures 13(b)–13(i) present images of hepatic tissue from diabetic mice. In general, the liver histological sections from diabetic mice present the accumulation of fat vacuoles in hepatocytes, mild inflammatory cell infiltration, vascular dilatation and congestion, and varying degrees of fibrosis at the pericellular and perisinusoidal levels. In the central area, degenerative changes characterized by the vacuolation of hepatocytes in the central zone area of hepatic lobules. Furthermore, the treatment of diabetic mice with 500 mg/kg BW/day *M. oleifera* leaf (G4) or seed (G5) extract for 1 month or 3 months (G7 and G8) did not show a significant improvement in the histopathological changes present in the diabetic liver, although the infiltration of inflammatory cells was milder, especially in the leaf extracted-treated group (G4 and G7) (Figures 13(d) and 13(g)). Using the combination extract (G9) for 3 months resulted in less congestion and less emptying of hepatocytes (Figure 13(i)), indicating hepatic tissue recovery.

The above histopathological findings on the improved effect of *M. oleifera* treatment on the kidney, pancreas, and liver tissues agree with the biochemical results presented in Table 3, especially in the diabetic mice treated with the *Moringa oleifera* combination extracts for 3 months (G9) or for 1 month (G6).

## 4. Discussion

Medical plants are rich in secondary metabolites that have an antihyperglycemic effect owing to their ability to increase insulin production or inhibit the intestinal absorption of glucose [3, 24, 75, 76]. Various bioactive phytochemicals were found in analyzed extracts with respectable amount of phenolic content, flavonoids, anthocyanidin, and alkaloids. The present results were in parallel with the previous findings [77–79].

*M. oleifera* leaves, seeds, and other plant parts possess a wide range of pharmacological properties, including antioxidant, anti-inflammatory, antihyperlipidemic, and antidiabetic effects [30, 56, 76, 80–82]. Hence, there has been a significant research interest in examining and evaluating the potential antidiabetic efficiency and protective actions of *M. oleifera* in the prevention and management of diabetes [82, 83]. It has been used in Asian traditional medicine to treat diabetes [84–87]. Numerous studies have reported on the antidiabetic (hypoglycemic) properties of *M. oleifera* leaf extract [88–99], seed extract [100–108], root extract [109, 110], pod extract [57, 111], fruit extract [19, 112], and flower extract [113, 114]. The hypoglycemic effect of the leaf, seed, root, pod, fruit, and flower of *M. oleifera* extracts was variable in the experimental animal models of induced diabetes, according to the dose and duration of treatment. The most significant hypoglycemic effect of *M. oleifera* plant parts was observed in the leaf and seed extracts. However, no studies were found regarding the antidiabetic effect of the combined (mixture) leaf/seed extracts of *M. oleifera*. Therefore, the present study was conducted to investigate the antidiabetic and antioxidant effects of the combined leaf/seed methanol extract of *M. oleifera* in the alloxan-induced model of diabetes.

DM is a major global disease burden that threatens the health and life quality of affected individuals. It is one of the leading causes of morbidity and mortality in the world [115]. The incidence of diabetes has been continuously increasing worldwide; the International Diabetes Federation predicts that 783 million people will be affected by the year 2045 [5]. Diabetes is characterized by high levels of glucose in the blood (hyperglycemia) causing vascular complications in vital organs such as the pancreas, liver, kidneys, eyes, nerves, heart, and blood vessels. These diabetic complications are the result of the impaired metabolism of lipids, proteins, and carbohydrates [6, 116]. Lipid abnormalities include increased levels of low-density lipoprotein, cholesterol, triglycerides, and low levels of high-density lipoprotein. Hyperlipidemia increases the risk of cardiovascular diseases [6, 115–117]. In addition, excess glucose is converted into various compounds, which leads to the overproduction of free radicals and reactive oxygen species (ROS) such as hydroxyl radicals (HO), superoxide anions (O_2_^−^), and hydrogen peroxide (H_2_O_2_) [118, 119]. ROS cause the cellular breakdown of proteins and plasma membrane lipids, resulting in an increase in protein and lipid peroxidation products (e.g., PC, MDA, and NO [120–123]. MDA, NO, and PC are the most common oxidative stress biomarkers that reflect tissue damage. Creatinine is also a protein breakdown product, and its elevation is associated with renal dysfunction [124, 125]. Concurrently and due to the compensatory mechanism in response to increased oxidative stress biomarkers, there is a decrease in antioxidant enzymes such as superoxide dismutase, catalase (CAT), glutathione peroxidase (GPx), and nonenzymatic antioxidant reduced glutathione (GSH) [126]. All of the aforementioned factors contribute to the development and progression of diabetic complications [127]. Furthermore, the loss of body weight in diabetes results from the degradation and catabolism of fats and proteins [128], and it is attributed to increased utilization of the body energy reserves accompanied by the absence of the regulatory hormone insulin [129].

The adverse complications of diabetes could be delayed and prevented by adequate glycemic and oxidative stress control, which is a major priority in the management of diabetes. Therefore, the assessment of the hypoglycemic and antioxidant effects of *M. oleifera* is of utmost importance to formulate a standard alternative natural and efficient medication for the treatment and management of diabetes.

Alloxan treatment is one of the most widely used methods of inducing diabetes in experimental animals [59]. It is a cytotoxic agent that selectively destroys the insulin-producing pancreatic *β*-cells of the islets of Langerhans when administered either intraperitoneally, intravenously, or subcutaneously. The destruction of *β*-cells results in the malfunction of insulin secretion and the reduction in glucose utilization by body tissues, which leads to hyperglycemia, liver injury, and kidney dysfunction [58, 59]. Consequently, hyperglycemia triggers an increase in the production of free radicals and ROS, causing oxidative stress and tissue damage. In this study, diabetes was successfully induced as all mice treated with alloxan developed hyperglycemia. Damage to renal and hepatic tissue was evident from the increased levels of creatinine [124, 125] and liver function enzymes AST, ALT, and ALP [94, 130, 131].

The results of the present study indicate that the leaf and seed extracts of *M. oleifera* have a reducing effect on glucose levels (i.e., antihyperglycemic or antidiabetic) in alloxan-induced diabetic mice. Therefore, this result confirms the antihyperglycemic properties of *M. oleifera* as previously reported by several researchers [58, 89, 105, 108]. Many studies have reported the positive effects of herbal medicines in the management of diabetes owing to their content of bioactive phytochemicals that are frequently implicated as having an antidiabetic effect [132, 133]. Plant secondary metabolites, including alkaloids, polyphenols, flavonoids, saponins, tannins, and terpenoids, have been shown to be responsible for the antihyperglycemic effect. The antihyperglycemic mechanism of herbal medicinal plants in diabetes-induced animal models could be due to their ability to restore pancreatic function by increasing insulin production and insulin sensitivity in peripheral tissues [3, 134] to enhance glucose uptake [135], as well as the inhibition of glucose transporter proteins in cell membranes by flavonoid glycosides [24, 133, 136]. Herbal medicinal plants also restore normal glucose metabolism in the liver by increasing the gene expression of glycogen storage enzymes [126, 134] and decreasing the gene expression of enzymes involved in gluconeogenesis [74, 125, 134]. They also reduce oxidative stress and enhance antiperoxidative activity to protect cells and tissues against ROS [137, 138].

The results of this study also confirm the antihyperlipidemic activity of leaf and seed extracts of *M. oleifera* by reducing cholesterol and triglyceride levels. These results are consistent with previous findings [139–142] that *M. oleifera* contributes to the maintenance of lipid homeostasis owing to the presence of high amounts of various bioactive phytochemicals such as isoquercitrin, chrysin-7-glycoside, and quercitrin [76]. The antihyperlipidemic activity of *Moringa* may be due to the extract inhibiting lipogenesis by activating the AMPK signaling pathway [143]. Additionally, the leaf and seed extracts of *M. oleifera* increased the body weight of alloxan-induced diabetic mice compared with nondiabetic control mice, probably due to the reversal of gluconeogenesis. This result agreed with previous findings [88, 134].

Furthermore, *M. oleifera* leaf and seed extracts improved the antioxidant status of tissues, reduced lipid peroxidation, and inhibited oxidative damage owing to the presence of various types of antioxidant compounds, such as ascorbic acid, flavonoids, phenolics, and carotenoids [142–145]. However, the leaf extract resulted in higher free radical scavenging activity as compared with the seed extract. These results were similar to previous findings [144, 145] and confirmed the antioxidant property of *M. oleifera*. Antioxidants derived from medicinal plant sources have attracted more attention as free radical scavengers because they protect against ROS-induced oxidative stress damage and regulate the oxidative complications of diabetes [25, 146]. Currently, natural antioxidants are used as supportive therapy in the management and treatment of diabetes [147–149].

Moreover, the results of this study showed the highly potent antihyperglycemic effect of the *M. oleifera* combined extract in alloxan-induced diabetic mice compared with their individual effects. The daily oral administration of the *M. oleifera* combined extract significantly decreased blood glucose levels, other biochemical parameters such as liver function enzymes (ALT, AST, and ALP), creatinine, cholesterol, triglycerides, and oxidants markers (MDA, NO, and PC), with a concurrent increase in antioxidant biomarkers, specifically CAT and GSH, and this improvement was significantly higher compared with the administration of leaf or seed extracts only. This improvement was also confirmed by histopathological examination and recovery of the pancreas, liver, and kidney. *M. oleifera* protected and revitalized the pancreatic tissue, islets of Langerhans, liver tissue, and kidney tissue. The antidiabetic, antilipidemic, and antioxidant protective effects of the *M. oleifera* combined extract were persistently maintained throughout the study period (i.e., short term, 28 days, or long term, 3 months). To the best of our knowledge, this is the first study to report on the antidiabetic, antioxidant, and antihyperlipidemic effects of the combined leaf/seed extract of *M. oleifera* in the alloxan-induced diabetes mouse model; the results are indeed promising.

## 5. Conclusion

The results of the present study have shown that the oral administration of the combined leaf/seed extract of *M. oleifera* reduced fasting blood glucose to normal levels much more effectively than if only the leaf or seed extract was administered. Additional studies on the combined extracts of different plant parts (e.g., leaf, seed, root, pod, fruit, and flower) of *M. oleifera* are recommended to find the most potent hypoglycemic and antioxidant combination. The experimental findings of this study appear to indicate a promising opportunity to support the development of a potent antidiabetic drug from *M. oleifera* for the treatment and management of diabetes.

However, only a few studies have investigated the antidiabetic therapeutic potential of *M. oleifera* in human subjects [150–155], and the results are inconsistent. Therefore, there is a need for clinical trials as almost all research has examined alloxan- or streptozotocin-induced models of diabetes. Additional clinical research on *M. oleifera* in human subjects would verify the protective effects and appropriate doses in humans, which will hopefully lead to the development of a natural antidiabetic therapy.

## Figures and Tables

**Table 1:** Experimental groups in this study.

Experimental group	Treatment
Group 1^∗∗^	Nondiabetic control mice without any treatment
Group 2^∗^	Diabetic control mice without any treatment
Group 3 ^∗^	Diabetic mice treated with 0.7 units/kg insulin/day for 1 month
Group 4 ^∗^	Diabetic mice treated with leaf extract (500 mg/kg BW/day) for 1 month
Group 5 ^∗^	Diabetic mice treated with seed extract (500 mg/kg BW/day) for 1 month
Group 6 ^∗^	Diabetic mice treated with mixture of leaf and seed extracts (500 mg/kg BW/day) (ratio 2L : 1S) for 1 month
Group 7^†^	Diabetic mice treated with leaf extract (500 mg/kg BW/day) for 3 months
Group 8^†^	Diabetic mice treated with leaf extract (500 mg/kg BW/day) for 3 months
Group 9^†^	Diabetic mice treated with mixture of leaf and seed extracts (500 mg/kg BW/day) (ratio 2L : 1S) for 3 months

L: leaves; S: seeds.  ^∗^Mice euthanized after 1 month. ^†^Mice euthanized after 3 months.

**Table 2:** Phytochemical analysis of extracts.

Phytoconstituents	Leaves	Seeds
Polyphenols	**+++**	**+**
Tannins	**+++**	**−**
Flavonoids	**++**	**+**
Alkaloids	**+++**	**++**
Saponins	**+**	**+**
Coumarins	**++**	**+++**
Terpenoids	**++**	**−**

+++ = copiously present; ++ = moderately present; + = slightly present; − = absent.

**Table 3:** The contents of total phenolics, total flavonoids, anthocyanidin, and alkaloids in crude aqueous leaf and seed extracts.

		Leaves	Seeds
Polyphenols	mg GAE/100 mg dw	22928 ± 11.4^a^	1173 ± 8.33^b^
Flavonoids	mg RE/100 mg dw	106.3 ± 0.6^a^	20.34 ± 0.4^b^
Anthocyanidin	mg CE/100 mg dw	93.8 ± 1.12^a^	8.99 ± 0.6^b^
Alkaloids	mg AE/100 mg dw	3.31 ± 0.02^a^	0.78 ± 0.017^b^

dw = dry weight; GAE = gallic acid equivalent; RE = rutin equivalent; CE = catechin equivalent; AE = atropine equivalent. Data are presented as mean value ± standard deviation (SD) of triplicate readings (*n* = 3). ^a,b^Means with different superscript letters in the same row differ significantly (*P* < 0.0001).

**Table 4:** Levels of biochemical parameters in diabetic mice treated for 3 months with 500 mg/kg BW/day *Moringa oleifera* leaf (G7), seed (G8), and combination (G9) extracts as compared with the percentage (%) change, decrease (▼) or increase (▲), in the diabetic control group (G2)^†^ and diabetic mice treated for 1 month with *Moringa oleifera* leaf (G4)^∗^, seed (G5)^◊^, and combination (G6)^‡^ extracts.

Biochemical parameter	Experimental mice groups treated with *Moringa oleifera* for 3 months					
	G7		G8		G9	
	Value	%	Value	%	Value	%
TG	136.67 ± 11.06	19%^∗^ 23%^†^	174.00 ± 9.54	8%^◊^ 2%^†^	129.00 ± 5.57	16%^‡^ 28%^†^
CT	213.33 ± 6.81	8%^∗^ 16%^†^	243.67 ± 8.02	13%^◊^ 4%^†^	203.33 ± 7.77	9%^‡^ 20%^†^
Creatinine	0.76 ± 0.07	22%^∗^ 45%^†^	1.29 ± 0.03	36%^◊^ 7%^†^	0.54 ± 0.07	33%^‡^ 61%^†^
ALP	121.33 ± 4.51	12%^∗^ 49%^†^	237.67 ± 5.51	4%^◊^ 0.4%^†^	117.67 ± 6.51	9%^‡^ 51%^†^
AST	184.00 ± 6.56	14%^∗^ 26%^†^	237.67 ± 5.51	12%^◊^ 5%^†^	170.67 ± 6.03	16%^‡^ 32%^†^
ALT	116.67 ± 8.08	14%^∗^ 24%^†^	147.67 ± 7.02	12%^◊^ 4%^†^	111.67 ± 12.86	16%^‡^ 27%^†^
MDA	0.68 ± 0.08	63%^∗^ 55%^†^	1.32 ± 0.43	43%^◊^ 12%^†^	0.58 ± 0.19	28%^‡^ 61%^†^
NO	7.29 ± 1.16	27%^∗^ 41%^†^	12.14 ± 1.23	33%^◊^ 1.3%^†^	4.97 ± 0.75	33%^‡^ 59%^†^
PC	5.16 ± 1.07	43%^∗^ 53%	10.26 ± 4.25	21%^◊^ 6%^†^	5.03 ± 0.87	17%^‡^ 55%^†^
GSH	87.28 ± 7.16	14%^∗^ 30%^†^	70.02 ± 10.57	40%^◊^ 13%^†^	113.07 ± 10.11	35%^‡^ 46%^†^
CAT	7.68 ± 1.10	17%^∗^ 44%^†^	5.24 ± 1.46	48%^◊^ 17%^†^	8.88 ± 1.17	27%^‡^ 51%^†^

TG: triglyceride; CT: cholesterol; ALP: alkaline phosphatase; AST: aspartate aminotransferase; ALT: alanine aminotransferase; MDA: malondialdehyde; NO: nitric oxide; PC: protein carbonyl; GSH: reduced glutathione; CAT: catalase. ^†^Compared with diabetic control.  ^∗^Compared with G4. ^◊^Compared with G5. ^‡^Compared with G6.

## Data Availability

Data used during the current study are available from the corresponding author.
